# Acinar‐Derived Organoids Reveal Stage‐Specific Control of Acinar Plasticity

**DOI:** 10.1002/advs.78350

**Published:** 2026-10-18

**Authors:** Yongfei Hou, Siyu Li, Chunying Li, Zhifan Lin, Dan He, Li Yang, Liping Fu, Qianyue Zhang, Tianping Zhou, Yao Yao, Xiaolei Yin

**Affiliations:** ^1^ Institute for Regenerative Medicine State Key Laboratory of Cardiovascular Diseases Shanghai East Hospital Frontier Science Center for Stem Cell Research School of Life Sciences and Technology Tongji University Shanghai China; ^2^ Medical Innovation Center Shanghai East Hospital School of Medicine Tongji University Shanghai China; ^3^ Department of Orthopedics Zhabei Central Hospital Affiliated to Tongji University Shanghai China

**Keywords:** acinar‐to‐ductal metaplasia, cell plasticity, oncogenic *Kras*, organoids, redifferentiation

## Abstract

Adult pancreatic acinar cells undergo ordered metaplastic conversion during injury and tumor initiation. Whether individual cells retain redifferentiation capacity after metaplastic expansion, and what signals each transition requires, remains unknown. Here, using organoids derived from single acinar cells, we show that expanded acinar‐lineage organoids can reacquire acinar features under stage‐specific culture conditions. We defined conditions that preserve acinar identity ex vivo and showed that epidermal growth factor (EGF)/EGF receptor (EGFR) signaling initiates conversion from this maintained state. The expansion trajectory recapitulates injury‐ and oncogene‐associated acinar‐to‐ductal metaplasia (ADM) programs in vivo. After expansion, the conditions that preserved acinar identity could not restore it; a staged regimen containing transient insulin‐like growth factor 1 receptor (IGF1R) inhibition and dexamethasone promoted redifferentiation. The Kras^G12D^;Trp53^R172H^;Pdx1‐Cre (*KPC*) genotype reduced EGFR dependence during initiation but destabilized post‐expansion redifferentiation; *Kras*‐pathway inhibition increased endpoint acinar‐marker expression. These findings demonstrate that acinar plasticity is organized by stage‐specific signals, and that the *KPC* genotype disrupts this control at specific transition points.

## Graphical abstract

An acinar‐derived organoid system maintains adult pancreatic acinar identity ex vivo, converts it along an ordered acinar‐to‐ductal‐like trajectory, and then partially restores it. Each transition carries its own signal requirement, with EGF/EGFR initiating conversion and a staged glucocorticoid–IGF1R switch driving redifferentiation. The KPC genotype lowers EGFR dependence at initiation and destabilizes recovery after expansion.

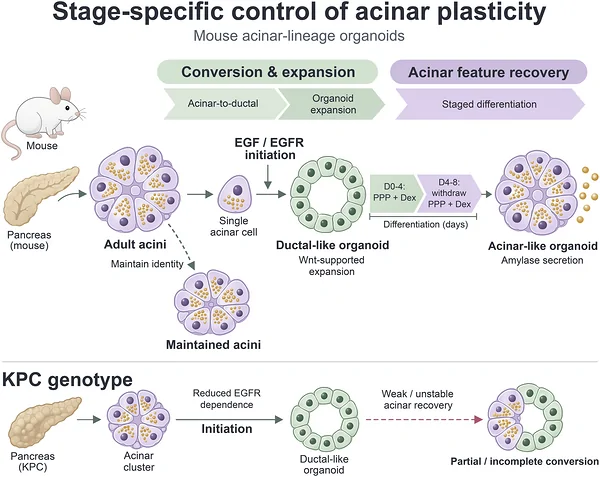

## 1 Introduction

Adult pancreatic acinar cells maintain their differentiated state through an active transcriptional program. *Ptf1a*–*Rbpjl*, *Mist1/Bhlha15* and *Nr5a2* sustain digestive‐enzyme expression and constrain metaplastic conversion [1, 2, 3, 4, 5, 6]. When injury or oncogenic signaling overrides this program, acinar cells undergo metaplastic conversion, suppressing secretory genes and acquiring ductal or progenitor features. In vivo, this conversion is termed acinar‐to‐ductal metaplasia (ADM) [7, 8, 9, 10, 11] and can resolve after acute injury as acinar identity is restored [12, 13]; under oncogenic *Kras*, however, metaplasia persists and progresses toward pancreatic ductal adenocarcinoma (PDAC) [7, 8, 9, 10, 12]. Sustained *Ptf1a* expression can counteract *Kras*‐driven neoplasia in vivo [6, 14], demonstrating that the acinar differentiated state is actively tumor‐suppressive. Whether individual acinar cells retain redifferentiation capacity after sustained metaplastic conversion, and what signals govern each stage of this process, remains unresolved.

Lineage‐tracing and single‐cell transcriptomic studies have mapped ADM as an ordered trajectory through intermediate states [12, 15, 16, 17, 18, 19] and implicated EGFR‐dependent signaling in *Kras*‐driven metaplasia [20, 21, 22]. In vivo, however, acinar tissue recovery after acute injury is attributed to replication of surviving acinar cells [12, 13], leaving unclear whether metaplastic cells themselves can reacquire acinar identity. Moreover, because identity maintenance, conversion, and potential reversion occur simultaneously, the signals controlling each transition have not been individually isolated.

Ex vivo systems should allow stage‐specific signal control, but acinar cells lose their identity within days of isolation [23, 24, 25, 26, 27], leaving no stable baseline from which to test conversion. Several complementary platforms address parts of this problem: duct‐ or progenitor‐derived organoids expand ductal cells long‐term [28, 29, 30, 31, 32]; pluripotent‐derived organoids generate acinar and ductal fates from developmental precursors [33, 34, 35]; and adult human acinar organoids model cancer onset from sorted acinar cultures [36, 37]. Each captures a specific aspect of acinar plasticity, yet the full trajectory, from a preserved acinar starting state through metaplastic expansion to post‐expansion redifferentiation, has not been achieved within a single system where signals at each transition can be independently controlled.

Here we recapitulate metaplastic conversion and redifferentiation in acinar‐derived organoids and define the signals controlling each transition. We first establish conditions that preserve adult acinar identity ex vivo and show that EGF/EGFR signaling initiates metaplastic conversion from this maintained state. Single acinar cells expand into organoids that traverse an ordered metaplastic trajectory aligned with pancreatitis‐ and *Kras*‐driven ADM in vivo. After expansion, organoids redifferentiate toward acinar identity when provided with transient inductive signals beyond those that sustain freshly isolated acinar cells. The *KPC* genotype promotes conversion while destabilizing post‐expansion redifferentiation. Together, these experiments establish a system for examining stage‐specific control of acinar plasticity.

## 2 Results

### 2.1 Defined Conditions Preserve Acinar Identity and EGF/EGFR Signaling Initiates Metaplastic Conversion

We first asked whether freshly isolated adult acinar cells could maintain acinar identity under defined ex vivo conditions, as acinar cells typically lose their identity within days of isolation [23, 24, 25, 26]. To track acinar‐cell fate, we used *Mist1‐CreERT2*; *Rosa26‐tdTomato* mice, in which tamoxifen‐induced recombination irreversibly labeled Mist1‐positive acinar cells with tdTomato [38]; 99.5% of Amylase‐positive acinar cells were labeled in vivo (Figure 1A,B and Figure S1A). We screened for conditions that could counter this identity loss, testing factors that promote cell survival, suppress ductal conversion, and maintain acinar gene expression. Candidates were selected from an available small‐molecule library to perturb major signaling axes and were advanced when they retained secretory‐enzyme expression without inducing ductal or proliferation genes.

**FIGURE 1:**
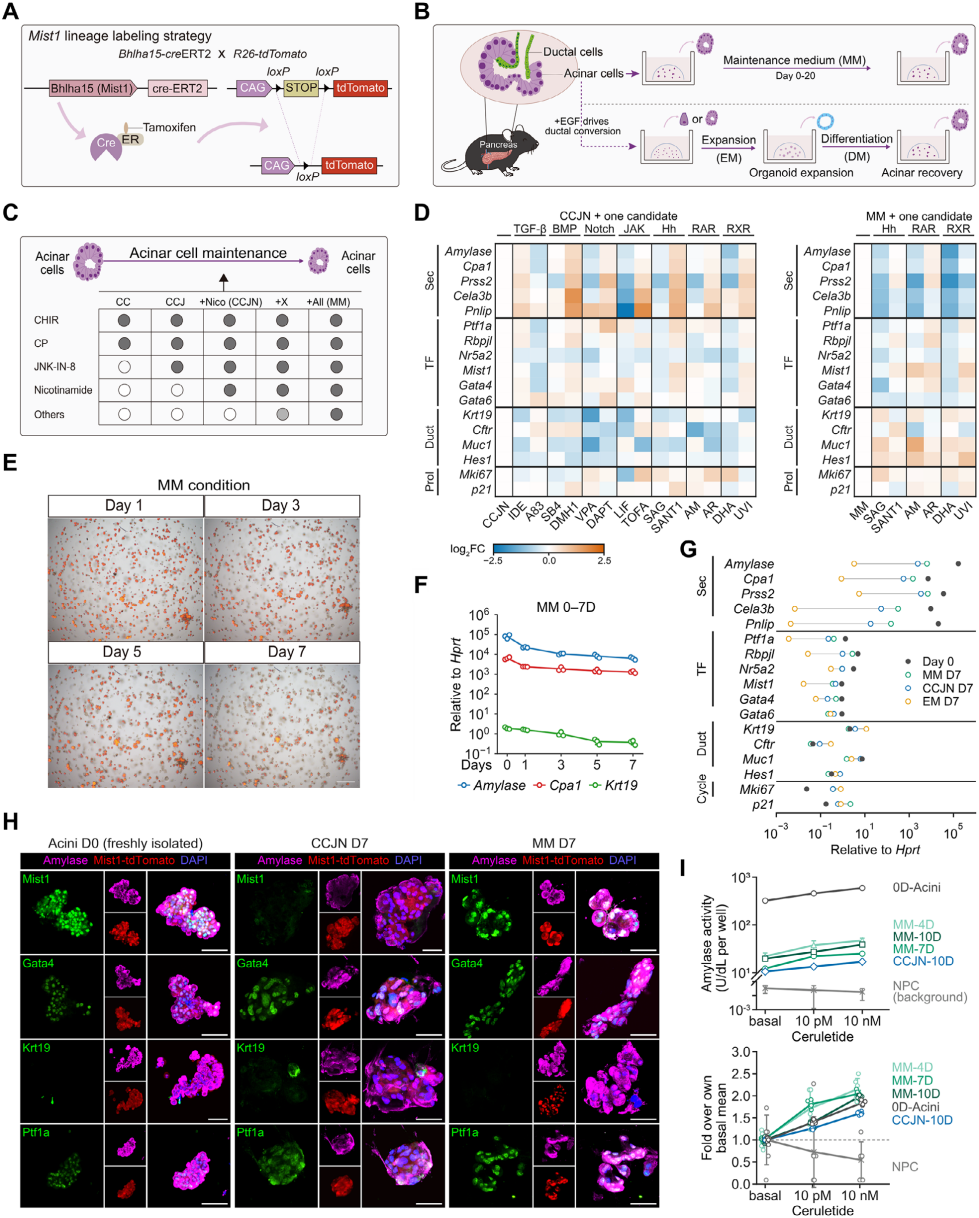
In vitro acinar maintenance. (A) Genetic scheme for *Bhlha15(Mist1)‐CreERT2*; *Rosa26‐tdTomato* lineage tracing of adult acinar cells. (B) Schematic workflow for acinar cell maintenance and generation of single‐cell‐ or cluster‐derived organoids and subsequent redifferentiation. Fresh acini were maintained in maintenance medium (MM) for over 20 d; single acinar cells or acinar clusters were cultured in ECCA expansion medium (EM) for 7 d and then transferred to differentiation medium (DM). (C) Stepwise schematic of the direct‐maintenance screen logic used for the condition comparisons in panel D and Figure S1C. (D) Gene expression after 7 d in CCJN supplemented with individual candidates (left) or in MM supplemented with additional candidates (right), shown as log2 fold change relative to the corresponding backbone control (*n* = 3 wells). (E) Representative morphology of acini during 7 d in maintenance medium. Scale bars: 500 µm. (F) Expression analysis of *Amylase*, *Cpa1*, and *Krt19* during 7 d of acinar maintenance in MM. Data are presented as mean ± SD (*n* = 3 mice). (G) Expression relative to *Hprt* in freshly isolated acini (day 0, approximately 2 hours after dissection) and after 7 d in MM, CCJN, or EM. Each point is the group mean (*n* = 3 wells). (H) Immunofluorescence of freshly isolated acini and of acini cultured for 7 d in CCJN or MM, stained for Mist1, Gata4, Krt19, or Ptf1a together with Amylase and the lineage reporter. For each antibody, acquisition and display settings were identical across the three states. Scale bars: 50 µm. (I) Amylase secretion in response to ceruletide across the indicated states and doses, including neural progenitor cells (NPC), an unrelated cell type included to define the background of the assay. Top, activity per well; bottom, activity relative to the basal mean of each state. Data are presented as mean ± SD; *n* = 2 samples per condition, each assayed in triplicate, and points are individual measurements.

CHIR‐99021 was included to probe Wnt signaling, given its role in pancreatic acinar specification and plasticity [10, 35], and CP‐673451 to test whether platelet‐derived growth factor receptor (PDGFR) inhibition supported maintenance. JNK‐IN‐8 was tested to reduce stress‐associated c‐Jun N‐terminal kinase (JNK) signaling, and nicotinamide because of its reported suppression of acinar dedifferentiation in culture (Table S2). A83‐01 and IDE1 were tested for transforming growth factor β/SMAD‐associated regulation, and SB4 and DMH1 for bone morphogenetic protein pathway activation and inhibition. DAPT and valproic acid tested Notch and histone deacetylase inhibition, respectively. Human LIF and tofacitinib tested cytokine/JAK–STAT signaling, SAG and SANT1 tested Hedgehog pathway modulation, and AM580, AR7, docosahexaenoic acid, and UVI‐3003 tested retinoid‐associated regulation. The rationale and supporting references for each candidate are provided in Table S2.

This screen identified a defined maintenance medium (MM) containing nicotinamide, JNK and PDGFR inhibitors, and the Notch inhibitor DAPT (Figure 1C,D and Figure S1B–D). Across secretory‐enzyme, acinar transcription factor, ductal, and proliferation genes, candidates added to the CCJN backbone or to MM produced distinct signatures, including additions that conferred no benefit (Figure 1D). Fresh acinar clusters cultured in MM preserved compact multicellular morphology for 7 d (Figure 1E). *Amylase* and *Cpa1* dropped soon after plating but then stabilized, whereas *Krt19* remained low throughout (Figure 1F). Across the same genes, MM‐maintained cells retained higher secretory‐enzyme and acinar transcription factor expression at day 7 than CCJN‐ or EM‐cultured cells (Figure 1G). Immunofluorescence at day 7 confirmed strong Amylase and tdTomato signal with minimal Krt19 staining (Figure 1H). MM‐maintained acini also secreted amylase and responded to ceruletide in a dose‐dependent manner for at least 10 d, whereas neural progenitor cells, included as an unrelated cell type, did not (Figure 1I). MM thus preserved acinar identity for at least 7 d.

Because EGF has been linked to ADM initiation in vivo [20, 22], and MM notably lacks it, we next asked whether EGF addition would be sufficient to disrupt this maintained state. Addition of EGF to MM cultures over 7 d reduced *Amylase* and *Cpa1* and increased *Mki67*, with higher mean *Krt19* and *Cftr* expression (Figure 2A). Gefitinib co‐treatment opposed these changes (Figure 2A,B). Over 20 d, EGF‐treated cultures showed sustained elevation of ductal and proliferative markers, whereas MM‐maintained cultures preserved stable acinar‐gene expression (Figure S1E). Acinar clusters cultured under 10% FBS or EGF + bFGF (EF) conditions, both of which contain EGF‐family ligands, also underwent rapid ductal conversion within 7 d (Figure S1F,G). These results show that EGF/EGFR signaling is sufficient to convert maintained acinar cultures toward a ductal state, and that acinar identity can be preserved under defined conditions that lack EGF.

**FIGURE 2:**
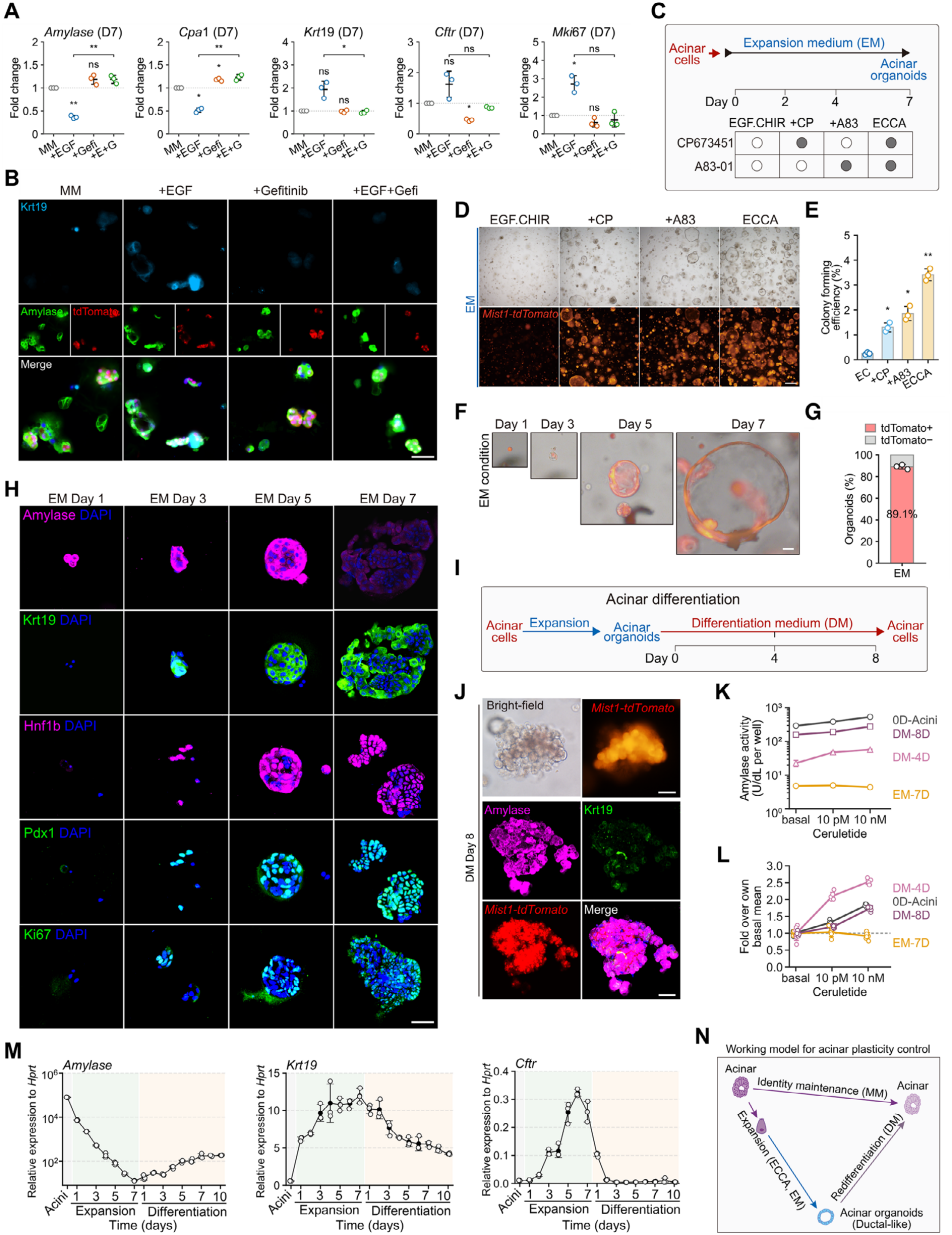
Culture of expandable acinar‐derived organoids that retain competence for acinar redifferentiation. (A) Expression analysis of *Amylase*, *Cpa1*, *Krt19*, *Cftr*, and *Mki67* in MM‐maintained acini and conditions treated with EGF, gefitinib, or both at day 7. Values are normalized to MM within each mouse. Data are presented as mean ± SD (n = 3 mice). Paired t tests with Holm correction were used for EGF versus MM, gefitinib versus MM, and EGF + gefitinib versus EGF within each gene. *P <.05; **P <.01; ***P <.001; ****P <.0001; ns, not significant. (B) Immunofluorescence of acinar cells cultured in MM or treated with EGF, gefitinib, or both at day 7, stained for Amylase and Krt19 together with the lineage reporter. EGF treatment promoted Krt19 expression, consistent with acinar‐to‐ductal metaplasia. Scale bars: 50 µm. (C) Schematic overview of culture conditions used to compare EC, EC + CP, EC + A83, and ECCA. (D) Representative bright‐field and tdTomato images of organoids generated under the conditions shown in panel C at day 7. *Scale bar*: 500 µm. (E) Colony‐forming efficiency under the conditions shown in panel C, calculated using the actual number of cells seeded. Data are presented as mean ± SD (*n* = 3 mice). Paired t tests with Holm correction were used for the three comparisons against EC. *P <.05; **P <.01. (F) Representative bright‐field and tdTomato images of a lineage‐traced single acinar cell during 7 d of expansion, showing progressive formation of a large cystic organoid. *Scale bar*: 50 µm. (G) Fraction of tdTomato‐positive and tdTomato‐negative organoids after 7 d of expansion. Points represent individual mice (*n* = 3 mice). (H) Immunofluorescence of organoids at days 1, 3, 5, and 7 of expansion stained for Amylase, Krt19, Pdx1, Hnf1b, and Ki67. *Scale bar*: 50 µm. (I) Schematic overview of 7 d of organoid expansion followed by 8 d of redifferentiation in DM. (J) Representative bright‐field and immunofluorescence images of redifferentiated organoids after 8 d in DM, showing compact grape‐like morphology and tdTomato, Amylase, and Krt19 staining. Scale bars: 500 µm for bright‐field and 50 µm for immunofluorescence. (K) Amylase secretion in response to ceruletide across the indicated states and doses. Data are presented as mean ± SD; *n* = 2 samples per condition, each assayed in triplicate. (L) Amylase secretion from the data in panel K relative to the basal mean of each state. Points are individual measurements. The dashed line indicates a fold change of 1.0. (M) qRT‐PCR analysis of *Amylase*, *Krt19*, and *Cftr* across 7 d of organoid expansion and 10 d of redifferentiation. Data are presented as mean ± SD (*n* = 3 wells). (N) Working model for acinar plasticity control. Acinar cells can be maintained long‐term in a defined maintenance medium or induced to proliferate, forming ductal‐like acinar‐derived organoids that subsequently redifferentiate toward an acinar identity.

### 2.2 Acinar Cells Retain Redifferentiation Competence after Metaplastic Expansion

Single acinar cells did not form organoids under 10% FBS or EF conditions (Figure S2A–C). Since EGF drives metaplastic conversion, we used it as the basis for an expansion medium and screened for additional factors, identifying ECCA: EGF combined with the Wnt agonist CHIR‐99021 for proliferative support, plus CP‐673451 and A83‐01, which improved organoid formation efficiency (Figure 2C–E). Fresh acini were dissociated to single cells and cultured in ECCA for 7 d. Single tdTomato‐positive acinar cells progressed to small spheroids and then to large cystic organoids, with increased organoid size after day 3 and predominantly large cystic organoids by day 7 (Figures 2F and S2D,E). At this point, 89.1% of organoids were tdTomato‐positive, indicating that most arose from lineage‐labeled acinar cells (Figure 2G). Given the near‐complete in vivo labeling, the tdTomato‐negative organoids are unlikely to be explained by incomplete recombination alone, although their origin was not resolved. The expanded organoids expressed Krt19, Pdx1, and Hnf1b with residual Amylase and tdTomato labeling (Figures 2H and S2F). Organoids also formed at seeding densities down to 100 cells per well, a 40‐fold dilution at which individual organoids are widely separated, consistent with formation from single cells rather than by aggregation (Figure S2G). The tdTomato‐negative organoids remained cystic after transfer to DM and redifferentiated far less efficiently than tdTomato‐positive organoids (Figure S2G,H). The same difference was apparent at the protein level: after 8 d in DM, a tdTomato‐positive organoid and an adjacent tdTomato‐negative organoid imaged in the same field showed Amylase staining in the lineage‐labeled organoid and little detectable Amylase in the unlabeled one (Figure S2I). Timed withdrawal of individual components (Figure S3A,B) showed that EGF acted primarily during the initial phase of organoid formation, whereas CHIR‐99021 supported organoid growth across a broader expansion window (Figure S3C–F). CP‐673451 and A83‐01 each increased organoid‐forming efficiency beyond the minimal EGF + CHIR backbone, with the full ECCA combination producing the strongest expansion (Figures 2C–E and S3G). Withdrawal of any component during expansion also weakened subsequent acinar‐marker re‐expression after redifferentiation (Figure S3H,I).

We tested whether expanded organoids could redifferentiate toward acinar identity. After transfer to a differentiation medium (DM) that combined the maintenance medium with transient IGF1R inhibition and dexamethasone, cystic organoids compacted into grape‐like clusters, remained tdTomato‐positive, and showed increased Amylase with reduced Krt19 (Figure 2I,J). Redifferentiated organoids also regained amylase secretion and ceruletide responsiveness, which were absent at the end of expansion (Figure 2K,L). qRT‐PCR during expansion and after redifferentiation showed reciprocal marker changes: *Amylase* and *Cpa1* decreased during expansion and increased after redifferentiation, whereas *Krt19*, *Cftr*, and *Mki67* changed in the opposite direction; *Pdx1* also decreased during redifferentiation (Figures 2M and S2J). After redifferentiation, ECCA‐derived organoids showed higher *Amylase* and *Cpa1* re‐expression, whereas FBS‐ and EF‐derived organoids largely remained cystic (Figure S2K,L), indicating that the expansion conditions influence whether redifferentiation competence is retained.

When intact acinar clusters rather than single cells were used as starting material, cluster‐derived organoids also expanded in ECCA and formed compact grape‐like structures after redifferentiation (Figure S4A,B). At higher magnification, amylase was confined to the cytoplasm and excluded from the nucleus in all three states, appearing as discrete puncta in freshly isolated acini and as a more densely punctate cytoplasmic pattern after maintenance or redifferentiation (Figure S4C). These cultures showed higher *Amylase* and *Cpa1* re‐expression after redifferentiation than single‐cell‐derived cultures (Figure S4D). Acinar‐derived organoids maintained cystic morphology, tdTomato labeling, and cumulative growth through serial passage (Figure S4E,F). Passaged organoids still underwent acinar redifferentiation after transfer to DM, although this response weakened at later passages, with lower acinar‐marker re‐expression and higher residual *Krt19* (Figure S4G). Together, these results establish a defined system in which acinar identity can be maintained, converted through metaplastic expansion, and redirected toward acinar identity, although redifferentiation competence diminished with prolonged passage (Figure 2N).

### 2.3 Organoid Expansion Traverses an Ordered Acinar‐to‐Ductal Trajectory

To define how acinar‐derived cells progress during organoid expansion, we profiled freshly isolated acini and ECCA cultures collected at days 1, 3, 5, and 7 by single‐cell RNA sequencing (Figure 3A). Integrated analysis identified acinar, ADM‐like, and Ductal‐like populations as the main epithelial compartments, along with minor non‐epithelial and residual ductal clusters (Figure 3B and Figure S5A–D). Acinar‐lineage cells formed a continuum spanning these three populations (Figure 3C and Figure S5E), progressing from acinar cells at day 0, through an ADM‐like intermediate at day 3, to a predominantly Ductal‐like population by days 5–7 (Figure 3D–F). The ADM‐like population co‐expressed the acinar marker *Cpa1* and the duct‐associated marker *Krt19* (Figure 3E and Figure S5F). Potency analysis scored freshly isolated acinar cells as terminally differentiated and showed a transient increase in developmental potency in a small fraction of ADM‐like cells. The Ductal‐like endpoint, however, consisted predominantly of unipotent and oligopotent cells (Figure 3G), elevated relative to the acinar starting point without reaching a highly plastic progenitor state. Acinar and duct‐associated markers segregated along this continuum, with *Cpa1* confined to the acinar end and *Krt19*, *Muc1*, and *Cdh1* enriched toward the Ductal‐like terminus (Figure 3H).

**FIGURE 3:**
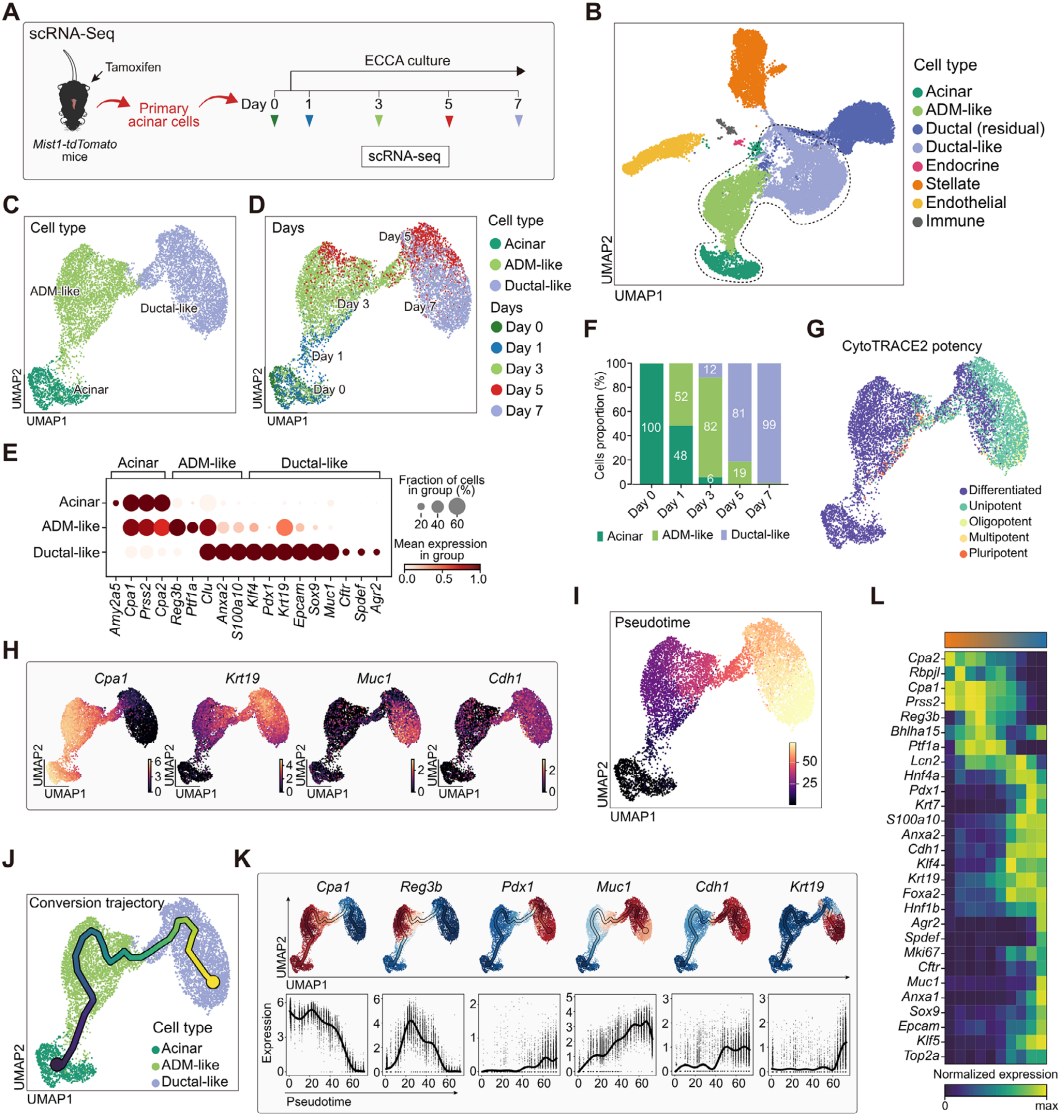
Acinar cells progress along an injury‐associated acinar‐to‐ductal continuum. (A) Schematic overview of scRNA‐seq sampling from freshly isolated acinar cells and cultures collected during the 7‐day expansion period. (B) UMAP plot showing major cell populations identified by integrated scRNA‐seq analysis. Cells outlined by the dotted line represent acinar‐lineage‐restricted cells. (C) UMAP plot showing acinar‐lineage‐restricted cells spanning Acinar, ADM‐like, and Ductal‐like populations. (D) UMAP plot showing sample origin within the acinar‐lineage‐restricted dataset. (E) Dot plot showing expression and percentage of cells expressing cell‐type‐specific markers across Acinar, ADM‐like, and Ductal‐like populations. Dot size indicates the percentage of cells expressing each gene, and color indicates normalized gene expression level. (F) Cellular composition analysis by cell type and sample across the acinar‐lineage‐restricted dataset. (G) CytoTRACE2 potency analysis across the acinar‐lineage‐restricted trajectory. (H) UMAP feature plots showing expression of acinar, ductal, and epithelial markers during acinar‐derived organoid expansion. Color intensity indicates normalized gene expression level. (I) Pseudotime analysis based on cell states and sampling time. (J) scFates analysis showing the inferred transition trajectory of acinar‐lineage cells. (K) scFates analysis of dynamic marker expression along pseudotime. Color intensity indicates normalized gene expression level. (L) Heatmap of marker‐gene expression along the pseudotime trajectory. Color intensity indicates normalized gene expression level.

Trajectory inference with scFates [39] placed acinar‐lineage cells along a single path from Acinar to Ductal‐like, with pseudotime increasing from day 0 to day 7 (Figure 3I,J and Figure S5G,H). Gene‐expression dynamics along this path showed progressive loss of acinar genes, a transient *Reg3b*‐high intermediate, and later accumulation of ductal genes including *Muc1* and *Krt19* (Figure 3K,L). Acinar‐derived organoid expansion thus followed an ordered acinar‐to‐ductal trajectory.

### 2.4 The Metaplastic Trajectory Recapitulates Conserved Injury‐ and Oncogene‐associated Regulatory Programs

To determine whether this trajectory corresponds to ADM programs in vivo, we constructed an integrated reference from two published single‐cell transcriptomic datasets: a pancreatitis ADM atlas (Ma et al.) [17] and an epithelial dataset from *Kras* ^G12D^ mice (Schlesinger et al.) [18]. The Ma et al. dataset contributed a complete acinar‐to‐ADM intermediate continuum, whereas the Schlesinger dataset provided oncogene‐driven metaplastic and ductal terminal states without a corresponding ADM intermediate (Figure 4A,B). On this integrated reference, Acinar, ADM‐like, and Ductal‐like populations mapped to contiguous regions along the acinar‐to‐metaplastic axis, with ADM‐like cells predominantly in the intermediate zone and Ductal‐like cells extending into metaplastic regions shared by both reference datasets (Figure 4C). Time‐resolved projection showed progressive movement from the acinar region into ADM and metaplastic reference regions from day 0 to day 7 (Figure 4D); both acinar and ductal markers retained their established polarity across the projection (Figure 4E).

**FIGURE 4:**
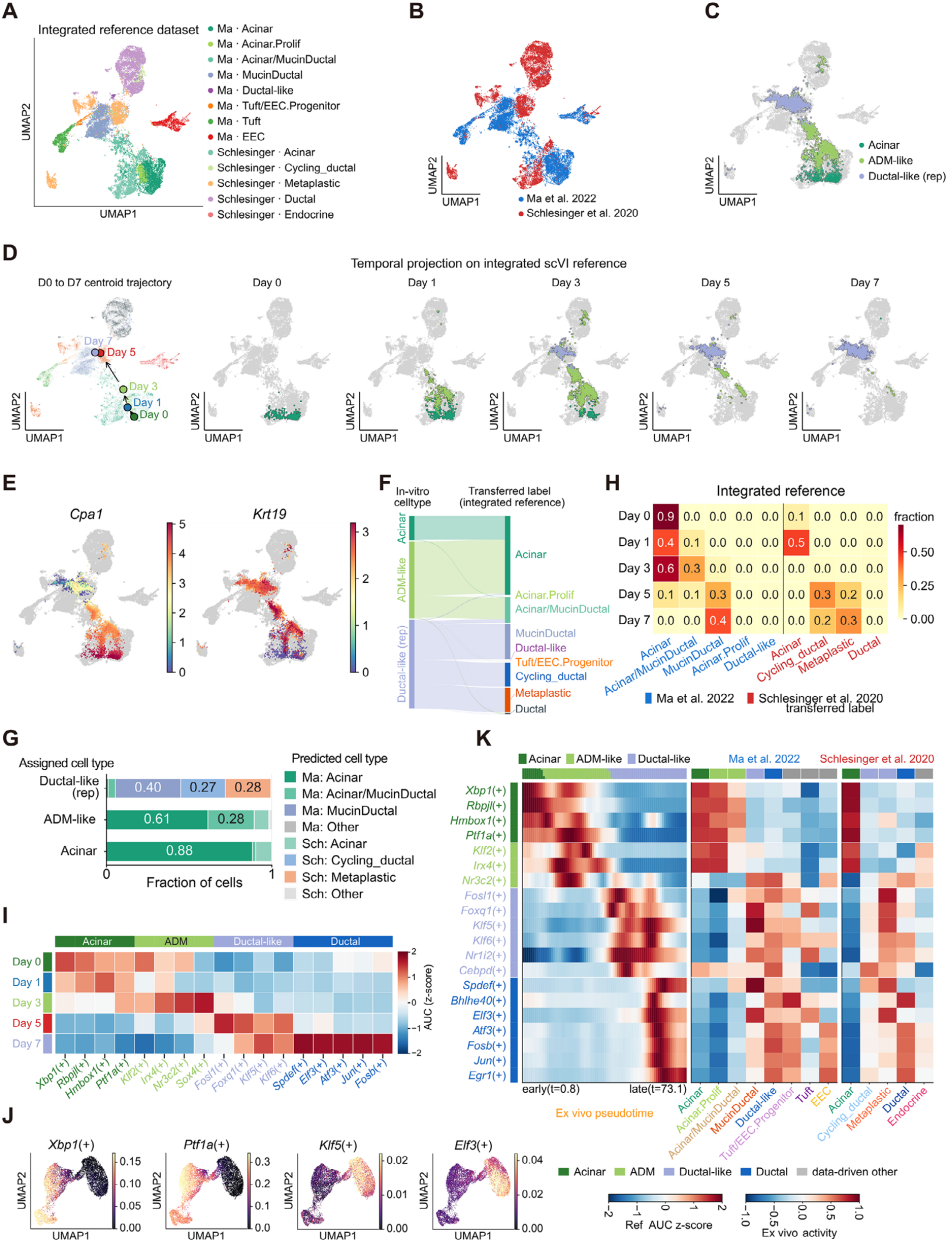
Ex vivo acinar‐lineage conversion maps to conserved ADM and metaplastic regulatory programs. (A) UMAP of integrated reference datasets combining the injury‐induced ADM scRNA‐seq dataset from Ma et al. (2022) and an oncogene‐induced ADM dataset from Schlesinger et al. (2020). Clusters from Ma et al. include Acinar, Acinar.Prolif, Acinar/MucinDuctal, MucinDuctal, Ductal‐like, Tuft/EEC.Progenitor, Tuft, and EEC; clusters from Schlesinger et al. include Acinar, Cycling_ductal, Metaplastic, Ductal, and Endocrine. (B) Integrated scVI reference UMAP colored by reference dataset origin. (C) Projection of ex vivo acinar‐lineage cells onto the integrated reference space, colored by in vitro cell state, revealing alignment with an acinar‐to‐ductal‐like axis. (D) Integrated scVI reference projection with D0–D7 centroid trajectory and per‐time‐point query overlays, showing progression from acinar and ADM‐like regions toward metaplastic and ductalized reference regions. (E) UMAP feature plots showing expression of selected markers on the integrated scVI projection. Color intensity indicates normalized gene expression level. (F) Query‐to‐reference identity mapping showing how ex vivo cell states resolve into integrated scVI reference labels. (G) Transferred‐label composition analysis showing how Acinar, ADM‐like, and Ductal‐like ex vivo states align with integrated scVI reference identities. (H) Time‐resolved transferred‐label analysis showing the accumulation of ADM, mucinous, metaplastic, and ductalized reference identities from D0 to D7. (I) Per‐sample activity of the 17 selected regulons from D0 to D7, grouped into acinar, ADM, ductal‐like, and ductal programs. Color intensity indicates z‐scored mean regulon activity. (J) UMAP feature plots showing activity of representative regulons across the ex vivo acinar‐lineage trajectory. Color intensity indicates regulon AUCell activity. (K) Data‐driven regulon cascade recovered by filtering dynamic regulons for pseudotime association and cross‐dataset concordance, independently of the selection in panel I. The resulting 20‐regulon order is shown across ex vivo pseudotime, Ma et al. (2022) cell states, and Schlesinger et al. (2020) cell states.

Label transfer confirmed these assignments: ADM‐like cells corresponded predominantly to acinar/ADM intermediates, whereas Ductal‐like cells distributed across MucinDuctal, Metaplastic, and Cycling_ductal labels (Figure 4F,G). Time‐resolved analysis showed progressive accumulation of ADM, mucinous, metaplastic, and ductal reference identities from day 0 to day 7 (Figure 4H). Parallel analyses on the Ma‐only reference confirmed these findings (Figure S6A–G).

To identify the regulatory programs driving these transitions, we performed regulon analysis, which revealed a temporal cascade: acinar regulons were active early, ADM‐associated regulons peaked at the intermediate phase, and ductal/metaplastic regulons dominated late (Figure 4I,J). An independent screen of transcription factors that changed along pseudotime, without prior annotation, recovered the same ordering in all three systems (Figure 4K), and program‐level activity of the selected regulons followed the same acinar‐to‐ductal progression across the three datasets (Figure S6H). Within the ex vivo data, the Ductal‐like population was not a uniform endpoint: some cells retained a MucinDuctal/Metaplastic regulatory program while others had progressed to a later ductal program, indicating that cells within the expanded population span different stages of the metaplastic trajectory, from the early ADM intermediates in the pancreatitis atlas to the late ductal states in the *Kras*‐driven dataset (Figure 4I,K). Organoid expansion was thus organized by a temporal cascade of transcription factor programs shared with injury‐ and oncogene‐driven ADM in vivo.

### 2.5 Acinar Redifferentiation Requires Transient Inductive Signals beyond Those That Maintain Acinar Identity

Having confirmed that the ex vivo trajectory recapitulates in vivo ADM programs, we next sought to identify the signals controlling redifferentiation. We first tested whether maintenance conditions were sufficient. MM preserved acinar identity in freshly isolated cells but did not restore acinar features after expansion. To identify candidate inductive signals, we examined pathway and gene‐level changes along the metaplastic trajectory. GSEA identified mTORC1 signaling as the top enriched Hallmark pathway among genes positively correlated with pseudotime, and gene‐level analysis showed that *Igf1r* and *Insr* increased toward the ductal‐like terminus whereas GR signaling components *Nr3c1* and *Fkbp5* peaked at an intermediate position and then declined at the ductal‐like terminus (Figure S7A–C). We therefore tested whether transient IGF1R inhibition with picropodophyllin (PPP) combined with dexamethasone (Dex) [40], added to the MM background, could initiate redifferentiation (Figure 5A). Under DM, expanded organoids compacted into grape‐like structures, activated the *Mist1* reporter, and showed progressive Amylase increase with concurrent Krt19 loss over 8 d (Figure 5B,C). Secretory‐enzyme genes and the acinar transcription factors *Ptf1a*, *Rbpjl*, *Mist1*, and *Nr5a2* rose progressively while ductal genes declined (Figure 5D,E), and *Ptf1a*, *Gata4*, and *Mist1* protein became detectable from day 4 onward (Figure 5F), with Amylase increasing and Krt19 declining over the same period (Figure 5G).

**FIGURE 5:**
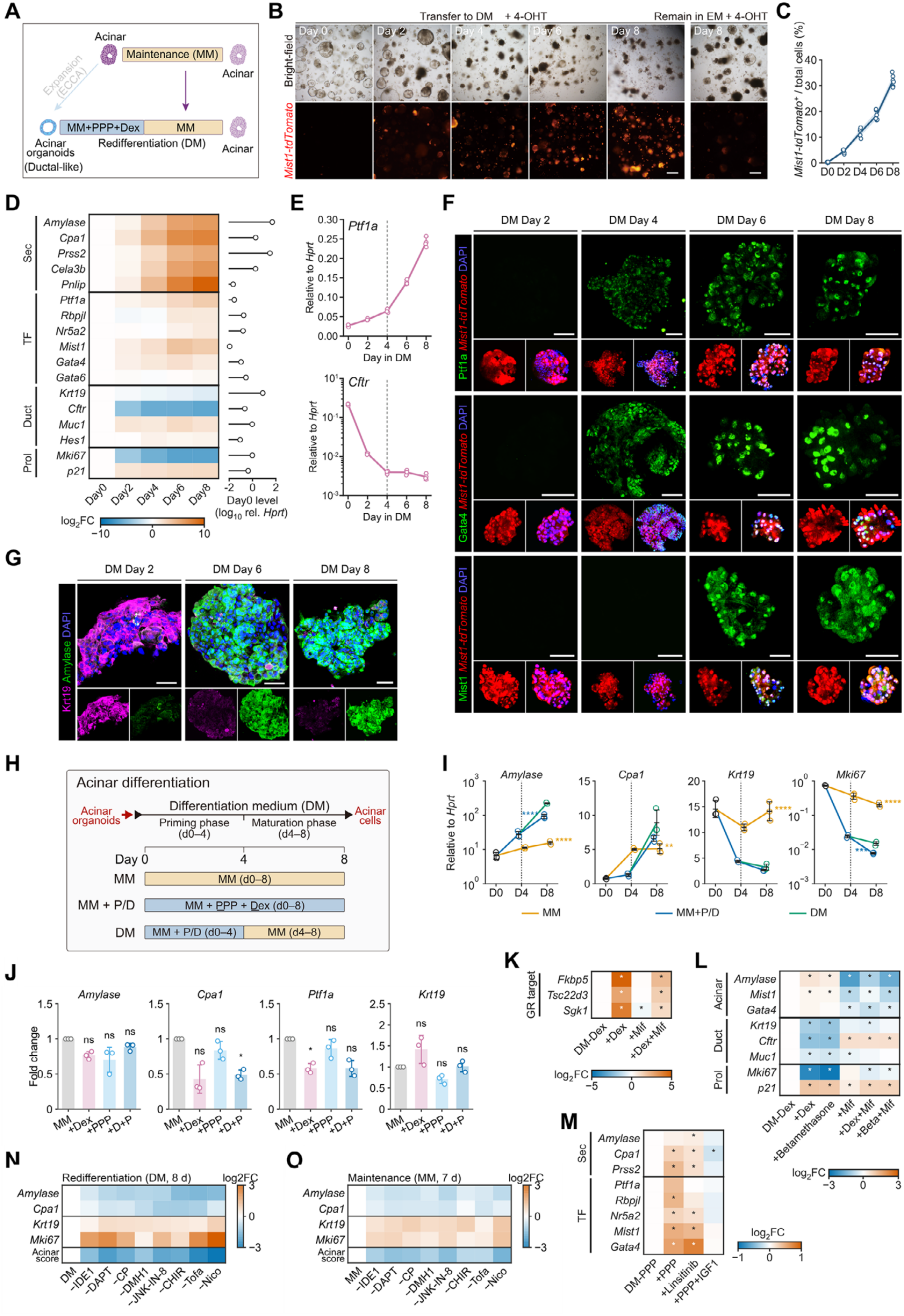
Acinar redifferentiation requires additional inductive signals beyond maintenance medium conditions. (A) Working model for acinar maintenance and acinar redifferentiation of ECCA‐cultured organoids. (B) Representative images showing organoid morphology and *Mist1*‐tdTomato expression at days 0, 2, 4, 6, and 8 after transfer to DM with 4‐OH tamoxifen induction, together with organoids kept in EM for 8 d under the same induction. Scale bars: 500 µm. (C) Quantification of *Mist1*‐tdTomato‐positive cells during redifferentiation. Data are presented as mean ± SD (*n* = 6 wells). (D) Gene expression across 8 d of redifferentiation in DM. Color indicates the log2 fold change relative to day 0; the right column shows the day‐0 level relative to *Hprt* (*n* = 3 wells). (E) Expression analysis of *Ptf1a* and *Cftr* across 8 d of redifferentiation in DM. Data are presented as mean ± SD (*n* = 3 wells). (F) Immunofluorescence of organoids at redifferentiation days 2, 4, 6, and 8 stained for Ptf1a, Gata4, or Mist1 together with the lineage reporter. Scale bars: 50 µm. (G) Immunofluorescence of organoids at redifferentiation days 2, 6, and 8 stained for Amylase and Krt19. Scale bars: 50 µm. (H) Schematic overview of redifferentiation conditions, including MM alone, continuous MM + P/D, and two‐step DM. (I) Expression analysis comparing MM, MM + P/D, and DM for *Amylase*, *Cpa1*, *Krt19*, and *Mki67* across 8 d of redifferentiation. Data are presented as mean ± SD (n = 3 wells). One‐way analysis of variance was used to calculate *P* values, followed by Dunnett's multiple‐comparisons correction. *P <.05; **P <.01; ***P <.001; ****P <.0001. (J) Acinar‐marker expression analysis in MM‐maintained acini treated with dexamethasone, PPP, or both. Values are normalized to MM within each mouse. Data are presented as mean ± SD (*n* = 3 mice). Paired t tests with Holm correction were used for the three treatment‐versus‐MM comparisons within each gene. *P <.05; ns, not significant. (K) Expression of the GR target genes *Fkbp5*, *Tsc22d3*, and *Sgk1* after 4 d in Step 1 DM lacking dexamethasone, or with dexamethasone, mifepristone, or both added back. PPP was retained during days 0–4. Color indicates the log2 fold change relative to DM lacking dexamethasone (*n* = 3 wells). Asterisks indicate Dunnett *P* <.05. (L) Gene expression at day 8 after dexamethasone, betamethasone, mifepristone, or the indicated combinations were added to dexamethasone‐free Step 1 DM during days 0–4 while PPP was retained. All groups received standard Step 2 DM during days 4–8. Color indicates the log2 fold change relative to DM lacking dexamethasone (*n* = 3 wells). Asterisks indicate Dunnett *P* <.05. (M) Gene expression at day 8 after PPP, linsitinib, or PPP + IGF1 was added to PPP‐free Step 1 DM during days 0–4 while dexamethasone was retained. All groups received standard Step 2 DM during days 4–8. Color indicates the log2 fold change relative to DM lacking PPP (*n* = 3 wells). Asterisks indicate Dunnett *P* <.05. (N) Summary heatmap of individual single‐component dropouts and the DM group after 8 d of redifferentiation. Color indicates the mean within‐mouse log2 fold change relative to the complete formulation (n = 3 mice). The acinar score is mean(log2FC of Amylase and Cpa1) minus mean(log2FC of Krt19 and Mki67). (O) Summary heatmap of individual single‐component dropouts and the MM group after 7 d of maintenance, presented as in panel N (*n* = 4 mice).

Redifferentiation was sensitive to the timing of PPP/Dex exposure. Both staged (first 4 d only) and continuous (8 d) PPP/Dex exposure reduced *Krt19* and *Mki67* relative to MM, but only the staged regimen produced strong late induction of *Amylase* and *Cpa1* (Figure 5H,I). During the maturation phase, withdrawing dexamethasone or PPP after day 4 raised Amylase and Cpa1, whereas dexamethasone add‐back lowered both genes and PPP add‐back lowered *Amylase* (Figure S7D). In freshly isolated acinar cells, by contrast, dexamethasone reduced *Ptf1a* and combined dexamethasone plus PPP reduced *Cpa1* (Figure 5J). The cellular response to PPP/Dex thus differed between freshly isolated acinar cells and expanded organoids.

To test whether the effects of the two staged cues were consistent with their proposed pathways, we used complementary pharmacological perturbations. Dexamethasone induced the canonical GR targets *Fkbp5*, *Tsc22d3*, and *Sgk1*, and mean expression was lower with mifepristone co‐treatment; a second agonist, betamethasone, reproduced the effect of dexamethasone on acinar‐gene expression, and mifepristone opposed the agonist response (Figure 5K,L). A structurally unrelated IGF1R inhibitor, linsitinib, reproduced the effect of PPP, and IGF1 addition opposed the PPP response (Figure 5M). Together, these results support roles for glucocorticoid receptor activation and IGF1R‐pathway inhibition within the staged redifferentiation regimen. Component‐withdrawal analysis showed lower mean acinar scores after removal of individual shared components of DM and MM, with nicotinamide removal producing the largest mean reduction during both redifferentiation and maintenance (Figure 5N,O and Figure S7E,F). These results support a common signal foundation for redifferentiation and maintenance, with additional staged cues for redifferentiation.

### 2.6 The *KPC* Genotype Reduces EGFR Dependence during Initiation and Destabilizes Post‐Expansion Redifferentiation

Having established that EGF acts primarily during the earliest phase of initiation (Figure S3A,C–F,H) and that staged PPP/Dex promotes redifferentiation (Figure 5H,I), we next asked how the *KPC* genotype alters both processes. We tested whether oncogenic *Kras* could bypass this EGF requirement, using acini from *KPC* mice carrying LSL‐*Kras* ^G12D/+^, *Trp53* ^R172H/+^, and *Pdx1‐Cre* alleles [41] (Figure 6A). In vivo, *KPC* pancreas contained Krt19‐positive epithelial foci within or adjacent to Amylase‐positive acinar tissue, including cells with overlapping Amylase and Krt19 signal, consistent with spontaneous acinar remodeling (Figure 6B). In vitro, *KPC* acini formed large cystic organoids that by day 7 showed strong Krt19, Pdx1, Hnf1b, and Ki67 with low residual Amylase (Figure 6C and Figure S8A).

**FIGURE 6:**
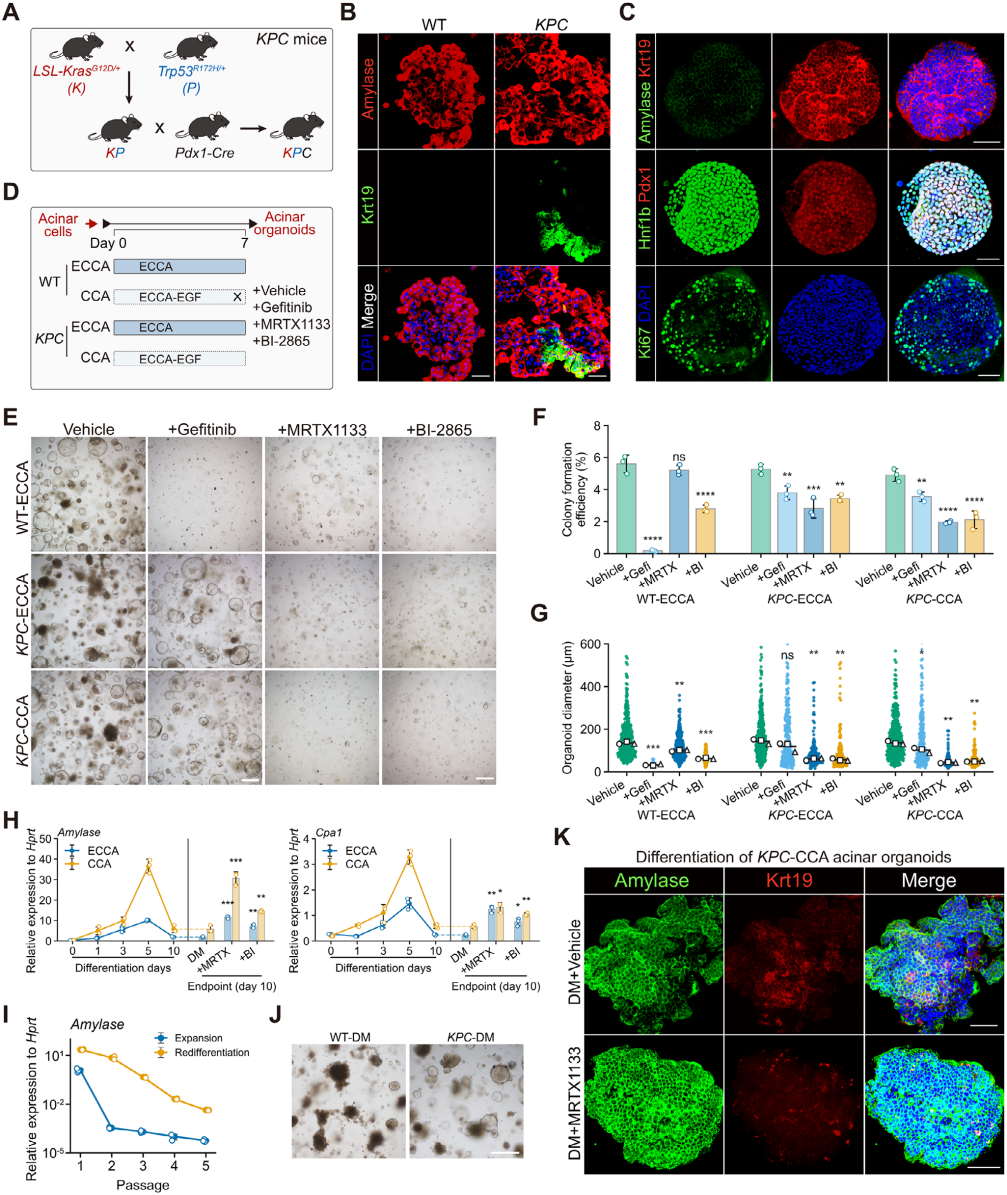
The *KPC* genotype reduces EGFR dependence during initiation and destabilizes post‐expansion redifferentiation. (A) Genetic scheme for the *KPC* model. (B) Immunofluorescence of WT and *KPC* pancreatic sections stained for *Amylase* and *Krt19*. Co‐expression of *Amylase* and *Krt19* indicates spontaneous acinar remodeling in the *KPC* pancreas. Scale bars: 50 µm. (C) Immunofluorescence of day‐7 *KPC* organoids stained for Amylase, Krt19, Pdx1, Hnf1b, and Ki67 in CCA. Scale bars: 50 µm. (D) Experimental design for testing EGFR dependence during organoid formation in WT and *KPC* acini under ECCA or CCA conditions with or without gefitinib, and for *Kras*‐pathway inhibition with MRTX1133 or BI‐2865 during expansion. (E) Representative bright‐field images of WT‐ECCA, *KPC*‐ECCA, and *KPC*‐CCA cultures treated with gefitinib, MRTX1133, or BI‐2865 during expansion. *Scale bar*: 500 µm. (F) Colony‐forming efficiency across the indicated conditions and treatments. Data are presented as mean ± SD (representative experiment with *n* = 3 wells per condition). One‐way analysis of variance was used to calculate *P* values, followed by Dunnett's multiple‐comparisons correction against the vehicle control of each culture model. **P <.01; ***P <.001; ****P <.0001. (G) Quantification of organoid diameter across the indicated conditions and treatments. Small points are individual organoids; open symbols are the median of each of three wells and the horizontal line is the mean of the three well medians. Unpaired Welch's t test on the well medians with Holm correction was used to calculate *P* values (*n* = 3 wells) against the vehicle control of each culture model. *P <.05; **P <.01; ***P <.001; ns, not significant. (H) Expression analysis of *Amylase* and *Cpa1* during redifferentiation of *KPC*‐ECCA and *KPC*‐CCA organoids, and at the day‐10 endpoint after *Kras*‐pathway inhibition with MRTX1133 or BI‐2865. Data are presented as mean ± SD (*n* = 3 wells). Unpaired Welch's t test was used to calculate *P* values. *P <.05; **P <.01; ***P <.001. (I) Expression analysis of *Amylase* before and after redifferentiation across passages in *KPC* organoids. Data are presented as mean ± SD (*n* = 3 wells). (J) Representative bright‐field images of WT and *KPC* organoids after redifferentiation in DM. Scale bar: 500 µm. (K) Immunofluorescence of vehicle‐ or MRTX1133‐treated *KPC*‐CCA organoids at the differentiation endpoint stained for Amylase and Krt19. Scale bars: 50 µm.

Wild‐type acini formed organoids in ECCA but not in CCA, the same medium without EGF, and gefitinib almost completely blocked organoid formation; by contrast, *KPC* acini generated cystic organoids in both ECCA and CCA, and gefitinib reduced but did not abolish organoid formation (Figure 6D–G). Within the *KPC* background, colony‐forming efficiency, organoid size, and gene‐expression profiles were comparable in ECCA and CCA across the 7‐day expansion period (Figure S8B–D). *KPC* acini also formed organoids under 10% FBS and EF conditions, but these cultures remained largely cystic and showed lower *Amylase* induction after redifferentiation (Figure S8E,F), reinforcing that the choice of expansion medium shapes subsequent redifferentiation independently of genotype. *KPC* acini thus formed organoids efficiently without exogenous EGF, with only partial sensitivity to EGFR inhibition. Because the *KPC* background combines *Kras* ^G12D^ with *Trp53* ^R172H^, this result is consistent with genetic evidence that p53 loss is sufficient to bypass EGFR dependence during *Kras*‐driven pancreatic tumorigenesis [22].

We next asked whether *Kras*‐pathway inhibition could suppress organoid outgrowth and increase endpoint acinar‐marker expression. During expansion, the *Kras* ^G12D^‐selective inhibitor MRTX1133 [42] and the pan‐*Kras* inhibitor BI‐2865 [43] each reduced cystic organoid formation in the *KPC* cultures, and both were associated with smaller organoid diameters across the three culture models (Figure 6E–G). Consistent with its genotype selectivity, MRTX1133 reduced colony formation in the *KPC* but not the wild‐type background (Figure 6F). In contrast to the sustained redifferentiation observed in wild‐type cultures, untreated *KPC* organoids showed only transient *Amylase* and *Cpa1* induction followed by decline, indicating that acinar redifferentiation was unstable in the oncogenic background (Figure 6H). This transient rebound was greater in CCA‐derived than in ECCA‐derived cultures, whereas *Krt19*, *Cftr*, *Pdx1*, and *Mki67* fell during early redifferentiation in both conditions (Figure 6H and Figure S8G). *Kras*‐pathway inhibition, particularly MRTX1133, increased endpoint *Amylase* and *Cpa1* in both ECCA‐ and CCA‐derived *KPC* cultures (Figure 6H). Redifferentiation in *KPC* organoids became progressively more unstable with serial passage, with acinar‐gene re‐expression weakening across passages (Figure 6I and Figure S8H,I). Consistent with this unstable redifferentiation, *KPC* organoids retained more cystic organoids after transfer to DM, whereas wild‐type cultures compacted into grape‐like clusters (Figure 6J). Immunofluorescence of MRTX1133‐treated *KPC*‐CCA organoids at the differentiation endpoint confirmed stronger Amylase and weaker Krt19 relative to vehicle‐treated controls (Figure 6K). The *KPC* background was therefore associated with unstable post‐expansion acinar redifferentiation. *Kras*‐pathway inhibition increased endpoint acinar‐marker expression at both the transcript and protein level, but not to wild‐type levels.

## 3 Discussion

Here we show that acinar‐derived organoids undergo metaplastic conversion and redifferentiation under stage‐specific signals. In vivo, ADM can resolve after acute injury [12, 13], although resolved injury leaves durable epigenetic memory that alters subsequent plasticity [44, 45]. This resolution, however, occurs within an intact microenvironment and has not been demonstrated at the single‐cell level. The present findings establish that expanded cells can re‐express acinar markers, although redifferentiation remained partial, possibly constrained by epigenetic changes accumulated during expansion.

The in vitro trajectory aligned with both pancreatitis‐ and *Kras*‐driven ADM at the level of cell‐state mapping and regulon activity, indicating that the core transcriptional program of ADM can be activated by defined signals ex vivo without the in vivo microenvironment. Critically, by the time cells reach the ductal‐like terminus, acinar transcription factor programs are largely silenced and ductal/metaplastic programs dominate, which may explain why redifferentiation did not fully restore the acinar transcriptional state. In this respect, the present system addresses an aspect not captured by prior acinar organoid cultures, which modeled metaplastic expansion but did not test post‐expansion redifferentiation [36, 37].

During both metaplastic conversion and redifferentiation, each transition separates into an initiation phase and a sustaining phase served by different signals. In the forward direction, EGF/EGFR signaling acts predominantly during the earliest phase of organoid formation, consistent with the established role of EGFR in ADM initiation [20, 22], whereas Wnt activation by CHIR‐99021 supports the subsequent expansion phase. This staged forward conversion, in which acinar cells dismantle secretory architecture, acquire ductal features, and re‐enter the cell cycle, is consistent with paligenosis, a conserved program by which differentiated secretory cells revert to a proliferative state through sequential checkpoints [46, 47]. The present findings identify the specific external signals that govern each step and extend this framework to include post‐expansion redifferentiation toward acinar identity. After expansion, maintenance signals alone do not initiate redifferentiation of expanded organoids. A transient PPP/dexamethasone‐containing first phase supported initiation of the transition, after which maintenance conditions sustained the regained acinar features; staged exposure during the first 4 d only produced stronger redifferentiation than continuous exposure, consistent with a role as an initiation cue rather than a maintenance factor. Notably, the same additions produced different marker responses in freshly isolated acinar cells, indicating context‐dependent responsiveness. Consistent with this context dependence, *Nr3c1* and *Fkbp5* expression varied along the metaplastic continuum, suggesting that cellular responsiveness to these signals differs between freshly isolated and post‐expansion states. This context‐dependent response raises the possibility that sustained ADM persistence, as seen under oncogenic *Kras*, reflects not a permanent loss of acinar competence but the absence of conditions that support reacquisition of acinar features after cell‐state change. Both IGF1R and the glucocorticoid receptor are pharmacologically accessible, and whether their modulation can promote ADM resolution in vivo warrants investigation.

The *KPC* background disrupts this stage‐specific organization at two points. During initiation, *KPC* acini formed organoids without exogenous EGF, placing EGFR upstream of the oncogenic lesion; because *Pdx1‐Cre* acts during pancreatic development and the *KPC* pancreas is already remodeled before culture, this reflects the *KPC* background as a whole rather than an adult acinar‐cell‐autonomous effect of oncogenic *Kras*, and the contributions of mutant *Kras* and mutant *Trp53* cannot be separated in this model. These findings may explain why ADM arises spontaneously in *Kras*‐mutant pancreas without overt injury [7, 8, 9]. At the second point, *KPC* organoids re‐expressed acinar markers only transiently during redifferentiation, in contrast to the sustained response of wild‐type cultures, and Kras‐pathway inhibition with MRTX1133 increased endpoint acinar‐marker expression without reaching wild‐type levels.

The ex vivo system described here captures the full arc of adult acinar plasticity and allows each transition to be dissected with defined signals. These data establish that expanded acinar‐lineage organoids retain redifferentiation competence under staged differentiation conditions, and that the KPC genotype is associated with facilitated conversion and less stable redifferentiation. This framework enables systematic testing of when and how differentiation‐based interventions can redirect expanded organoids toward acinar recovery.

### 3.1 Limitations

The system is mouse‐based and entirely ex vivo, lacking stromal, immune, vascular, and neural inputs. Redifferentiated organoids did not fully reconstruct the fresh adult acinar state. Secretory function was assessed only by amylase release and ceruletide responsiveness, and polarity and ultrastructure were not examined. Serial passage progressively weakened redifferentiation competence. The culture conditions likely capture only a subset of the metaplastic landscape observed in vivo [12, 17, 18]. Whether human acinar cells share these signal dependencies remains untested. Because viability, apoptosis, pERK/pMEK, and stage‐restricted inhibitor exposure were not assessed, the increased endpoint acinar‐marker signal cannot distinguish direct redifferentiation from altered proliferation or survival of ductal‐like organoids, or from selection or persistence of residual acinar‐like cells.

## 4 Materials and Methods

### 4.1 Mouse Strains

*Bhlha15(Mist1)‐CreERT2* mice (NM‐KI‐200080; Shanghai Model Organisms Center) were crossed with *Rosa26‐tdTomato* reporter mice (Jackson Laboratory, #007909) [38], and F1 offspring were used for acinar lineage tracing. *KPC* mice (NM‐KI‐210096; Shanghai Model Organisms Center) were used as a spontaneous PDAC model [41], with pancreata collected at approximately 8 weeks of age. Mice of both sexes were used. All animal procedures were approved by the Laboratory Animal Ethics Committee of Tongji University (TJAB04720101).

### 4.2 Acinar Cell Isolation

For lineage tracing, 8‐week‐old mice received tamoxifen in corn oil by oral gavage for 3 consecutive days (100 µL per dose; 15 mg mL^−1^). One week later, the pancreas was collected into ice‐cold DPBS, rinsed, minced, and digested in DMEM containing collagenase (0.15 mg mL^−1^), DNase I (0.1 mg mL^−1^), and penicillin‐streptomycin for 30 min at 37°C. The digest was gently pipetted to disperse residual fragments, briefly pelleted, and washed by repeated sedimentation in ice‐cold DPBS to enrich acinar clusters, based on established mouse acinar isolation approaches [48]. Each acinar isolation was prepared from a single mouse and supplied all conditions of a given experiment.

Acinar clusters were further dissociated in TrypLE for 5 minutes at 37°C to generate single cells. Single cells were seeded at 4000 cells per well, and cluster‐based cultures at approximately 800 clusters per well, in Cultrex RGF basement membrane extract (BME) droplets in 48‐well plates. After gel solidification, cultures were overlaid with medium containing 10 µm Y‐27632 and fed on day 4. For all analyses, day 0 denotes freshly isolated acinar cells collected immediately after digestion and purification and before plating, approximately 2 h after dissection.

### 4.3 Acinar Maintenance

For direct acinar maintenance, freshly isolated acinar clusters were embedded in BME and cultured in maintenance medium (MM), defined as the full differentiation base medium plus 10 µm Y‐27632. Medium was changed on day 4. For stepwise maintenance optimization, the reduced CCJ backbone contained CHIR‐99021, CP‐673451, and JNK‐IN‐8 on the basal medium; CCJN denotes CCJ plus nicotinamide. Where indicated, individual candidates were added to the specified backbone condition. Complete medium compositions are provided in Table S1, maintenance‐screen candidates in Table S2, and sources, identifiers, and working concentrations for proteins and small molecules in Table S3.

### 4.4 Organoid Expansion

Basal medium consisted of Advanced DMEM/F12 supplemented with B‐27, 10 mm HEPES, 2 mm GlutaMAX, penicillin‐streptomycin, and 1 mm N‐acetyl‐L‐cysteine. Unless otherwise indicated, expansion medium additionally contained 10% R‐spondin1 conditioned medium and EGF (50 ng mL^−1^), CHIR‐99021 (4 µm), CP‐673451 (0.25 µm), and A83‐01 (0.5 µm); this condition is referred to as ECCA. CCA denotes the same formulation without EGF. Established organoids were passaged from day 7 onward by dissociation into small clusters and re‐embedding in fresh BME at a 1:6 to 1:8 split ratio every 2 to 3 d.

### 4.5 Direct Differentiation in Organoids

Organoids were differentiated by direct medium switch. The standard two‐step protocol comprised a 4‐day Step 1 medium containing IDE1 (2 µm), CHIR‐99021 (4 µm), DMH1 (2 µm), DAPT (5 µm), dexamethasone (10 µm), picropodophyllin (PPP; 0.2 µm), JNK‐IN‐8 (2 µm), nicotinamide (1 mm), tofacitinib (2 µm), and CP‐673451 (0.25 µm), followed by a 4‐day Step 2 medium identical in composition except that dexamethasone and PPP were omitted. In experiments monitoring gene‐expression changes throughout the process, the differentiation was prolonged to day 10 (4 + 6). Where indicated, 4‐hydroxytamoxifen (4‐OH tamoxifen; 1 µm) was added during differentiation to activate the *Mist1*‐tdTomato reporter. For reporter‐reactivation experiments, mice were not exposed to tamoxifen before acinar isolation; 4‐OH tamoxifen was added only upon transfer to DM or to the matched EM control. For the maturation‐phase timing experiments (Figure S7D), all groups received picropodophyllin and dexamethasone during days 0–4; during days 4–8, each component was either added to standard Step 2 medium or withdrawn from continuous exposure, as indicated. Where individual components were tested (Figure 5K–M), Step 1 medium was prepared without dexamethasone or without picropodophyllin and single components were added back during days 0–4: dexamethasone (10 µm), betamethasone (5 µm), mifepristone (10 µm), or the indicated combinations to the dexamethasone‐free medium, and picropodophyllin (0.2 µm), linsitinib (1 µm), or picropodophyllin together with IGF1 (100 ng mL^−1^) to the picropodophyllin‐free medium; glucocorticoid receptor target genes were measured after 4 d and acinar, ductal, and proliferation genes after 8 d.

### 4.6 Immunostaining

Organoids were released from BME with DPBS, fixed in 4% paraformaldehyde for 30 min at room temperature, permeabilized in DPBS containing 0.5% Triton X‐100, 0.5% Tween 20, and 2% BSA, and incubated overnight at 4°C with primary antibodies in blocking buffer (0.1% BSA and 0.5% Tween 20 in DPBS). After washing, samples were incubated with fluorescent secondary antibodies (1:1000) for 1 h at room temperature and counterstained with DAPI. Images were acquired on an Olympus IX73, an Olympus FV3000 confocal microscope (IX83 stand; UPLAPO60XOHR 60×/1.5 NA oil‐immersion objective; 0.138 µm per pixel), or a Zeiss LSM880 microscope and analyzed in ImageJ [49]. For each antibody, acquisition settings were fixed on a reference sample and held constant across all samples within a panel, and images are displayed with identical brightness and contrast scaling. Settings differ between antibodies, so intensities are compared between states within a channel and not across channels or panels. The antibodies used in this study are listed in Table S4.

### 4.7 RNA Extraction and Reverse Transcription Quantitative PCR

Organoids were washed with ice‐cold DPBS and lysed in TRIzol. Total RNA was extracted according to the manufacturer's instructions; up to 1 µg RNA was reverse‐transcribed with NovoScript Plus Reverse Transcriptase, and qPCR was performed with ChamQ Universal SYBR qPCR Master Mix on a CFX384 system. Relative expression was calculated by a ΔCt method [50]. Primer sequences used for qRT‐PCR are provided in Table S5.

For the single‐component dropout screens, expression was reported as the mean within‐mouse log2 fold change relative to the complete formulation. The acinar score was calculated as mean(log2FC of *Amylase* and *Cpa1*) minus mean(log2FC of *Krt19* and *Mki67*).

### 4.8 Colony Formation Assay

Single acinar cells were plated at 4,000 cells per well in BME in 48‐well plates. After 7 d, colonies were fixed, stained with DAPI, and counted manually. Colony‐forming efficiency was calculated as colonies/plated single cells × 100%. Redifferentiation efficiency was scored morphologically as the fraction of organoids that compacted from a cystic to a grape‐like morphology after 8 d in DM; where organoids were classified by lineage label, tdTomato‐positive and tdTomato‐negative organoids were scored separately within the same wells.

### 4.9 Amylase Secretion Assay

Cultures were washed and incubated with DMSO, 10 pm ceruletide, or 10 nm ceruletide for 30 min at 37°C, and the supernatant was collected. Amylase activity was measured with an α‐amylase assay kit (Nanjing Jiancheng Bioengineering Institute, Cat#C016‐1‐2) according to the manufacturer's instructions: diluted supernatant was incubated with substrate for 7.5 min, the reaction was stopped with iodine solution, and absorbance was read at 660 nm. Samples comprised freshly isolated acini, acini maintained in MM for 4, 7, or 10 d, acini maintained in CCJN for 10 d, neural progenitor cells as an unrelated control, organoids at expansion day 7, and organoids at differentiation days 4 and 8. Supernatant was pooled from the wells of each condition, two samples were collected per condition, and each sample was assayed in triplicate. Activity is expressed per well, calculated from the measured concentration and the volume of pooled supernatant.

### 4.10 Single‐Cell RNA‐Sequencing Data Processing

Fresh acinar cells (day 0) and ECCA cultures harvested on days 1, 3, 5, and 7 were processed for single‐cell RNA‐seq on the DNBelab C4 platform (BGI). Vendor‐filtered count matrices were imported into R (v4.3.2) and converted to Seurat objects (Seurat v5.3.0) [51, 52]. Cells were quality‐filtered per sample on the basis of UMI counts, detected genes, mitochondrial fraction, nuclear fraction (intronic‐to‐total UMI ratio), and scDblFinder (v1.16.0) singlet classification [53]. Quality‐filtered cells were merged for downstream analysis.

Integrated analysis was performed in Python (v3.10.14) with Scanpy (v1.11.4) and BBKNN (v1.6.0) [54, 55]. Raw counts were normalized to 10 000 per cell and log‐transformed. The 2,000 most variable genes were selected per batch (Seurat v3 method), followed by principal‐component analysis, batch‐balanced nearest‐neighbor graph construction with BBKNN, UMAP embedding, and Leiden clustering at resolution 0.8 [56, 57]. Clusters were annotated as acinar, ADM‐like, residual ductal, ductal‐like, endocrine, stellate, endothelial, or immune on the basis of canonical markers.

For acinar‐lineage‐restricted analyses, tdTomato‐positive cells within the acinar, ADM‐like, and ductal‐like compartments were retained and re‐clustered with the same pipeline. Developmental potency was estimated per sample with CytoTRACE2 (v1.0.0) on raw counts (species = mouse) [58]. Trajectory inference was performed with scFates (v1.2.1) [39]: a principal curve was fit in PCA space, the root was assigned to the tip with the lowest mean sampling‐day index, and pseudotime orientation was validated by correlation with CytoTRACE2 potency scores.

### 4.11 Reference Mapping onto In Vivo ADM Datasets

Two published references were used: the Ma et al. pancreatitis atlas (GSE172380) [17] and the Schlesinger et al. *Kras* ^G12D^ epithelial dataset (GSE141017) [18]. For individual reference mapping, query and reference were restricted to shared genes, and cell‐type labels were transferred with Scanpy ingest. For the integrated reference, the two datasets were combined and batch‐corrected with scVI (scvi‐tools v1.3.1; 30 latent dimensions, negative‐binomial likelihood) [59]; query cells were projected onto the integrated reference space via ingest. For each projected cell, a pancreatitis‐versus‐*Kras* ^G12D^ neighborhood preference was computed as the pancreatitis fraction among its 10 nearest reference neighbors. Mapping confidence was quantified as the Shannon entropy of reference labels among each query cell's 10 nearest neighbors.

### 4.12 Transcription‐Factor Regulon Analysis

Gene regulatory networks were inferred with the SCENIC pipeline [60] using pySCENIC (v0.12.1) [61] and GRNBoost2 [62] on the acinar‐lineage subset (raw counts). Coexpression modules were pruned against two mouse cisTarget v10 motif databases (500 bp upstream / 100 bp downstream, and 10 kb upstream / 10 kb downstream of the TSS), retaining regulons with a normalized enrichment score ≥ 3.0; the regulons assigned to each of the four programs are listed in Table S6; per‐cell regulon activity was scored with AUCell. The same regulon definitions were applied to the Ma and Schlesinger reference datasets to enable cross‐dataset comparison; mean AUCell scores were z‐scored within each dataset before plotting. Dynamic regulons were defined as those whose activity correlated with pseudotime (Spearman |*ρ*| ≥ 0.2, Benjamini–Hochberg FDR ≤ 0.05), and the cross‐dataset cascade (Figure 4K) retained regulons with concordant ordering across all three datasets.

### 4.13 Statistical Analysis

Results are expressed as mean ± standard deviation (SD). The replicate unit is specified in each figure legend. For mouse‐level analyses, culture wells were averaged within each mouse and condition before comparison. Where n denotes wells, the data are from one isolation and comparisons describe variation within that preparation. Repeated assay measurements in Figures S1G and S7D were averaged within wells. Mouse‐level qPCR comparisons used log2 expression ratios relative to the matched control. Statistical tests and multiple‐comparison corrections are specified in the figure legends. All t tests were two‐sided, and *P* <.05 was considered statistically significant; ns, not significant. For organoid diameter, comparisons used well medians, except in Figure S2E, which used mouse means.

## Author Contributions

Conceptualization: Y.H., S.L., and X.Y. Data curation: Y.H., S.L., C.L., Z.L., D.H., L.Y., L.F., Q.Z., and X.Y. Formal analysis: Y.H., S.L., C.L., Z.L., D.H., L.Y., and X.Y. Funding acquisition: D.H., L.Y., and X.Y. Investigation: Y.H., S.L., C.L., Z.L., D.H., L.Y., L.F., Q.Z., and T.Z. Methodology: Y.H., S.L., C.L., Z.L., D.H., L.Y., L.F., Q.Z., and T.Z. Project administration: Y.Y. Resources: Y.Y. and X.Y. Supervision: X.Y. Visualization: Y.H., S.L., C.L., and X.Y. Writing – original draft: Y.H., S.L., and X.Y. Writing – review and editing: Y.H., S.L., C.L., Y.Y., and X.Y.

## Conflicts of Interest

The authors declare no conflicts of interest.

## Supporting information




**Supporting File**: advs78350‐sup‐0001‐SuppMat.docx.

**Supporting File**: advs78350‐sup‐0002‐SuppMat.xlsx.

## Data Availability

The raw sequence data reported in this paper have been deposited in the Genome Sequence Archive in the National Genomics Data Center, China National Center for Bioinformation / Beijing Institute of Genomics, Chinese Academy of Sciences (GSA: CRA040013). All data needed to evaluate the conclusions are present in the paper and/or the Supplementary Materials.
