# Supplementary material for: Acinar‐Derived Organoids Reveal Stage‐Specific Control of Acinar Plasticity
Source: Adv Sci (Weinh). 2026 Oct 18:e78350. Online ahead of print. doi: 10.1002/advs.78350 (PMC13677045; doi:10.1002/advs.78350)
Supplement: Supplementary file 1 [file ADVS-9999-e78350-s002.docx]

**Figure S1. In vitro acinar maintenance under defined conditions.**

**(A)** Immunofluorescence of pancreatic sections 7 days after tamoxifen induction, stained for Amylase, with quantification of *Mist1*-tdTomato-positive cells among Amylase-positive acinar cells. Data are presented as mean ± SD (n = 3 mice).

**(B)** Representative images showing the effects of JNK-IN-8 and nicotinamide addition on acinar maintenance. *Scale bar:* 500 μm.

**(C)** Expression analysis of *Mki67*, *Krt19*, *Cpa1*, and *Amylase* in fresh acini, CCJ, CCJN, CCJN supplemented with IDE1, DMH1, DAPT, or Tofa, and maintenance medium. Data are presented as mean ± SD (n = 3 wells). One-way analysis of variance was used to calculate *P* values, followed by Dunnett’s multiple-comparisons correction. ***P* < .01; ****P* < .001; *****P* < .0001; ns, not significant.

**(D)** Representative bright-field and tdTomato images of day-7 acinar cultures after adding individual components to the CHIR-99021 or CP-673451 backbone of the maintenance medium. *Scale bar:* 500 μm.

**(E)** Expression analysis of *Amylase*, *Cpa1*, *Ptf1a*, *Mist1*, *Krt19*, *Cftr*, and *Mki67* from day 2 to day 20 of acinar maintenance in MM or treated with EGF, gefitinib, or both. Data are presented as mean ± SD (n = 6 wells).

**(F)** Representative day-7 images of acinar cultures in MM, 10% FBS, or EF. *Scale bar:* 500 μm.

**(G)** Expression analysis of *Amylase*, *Cpa1*, *Krt19*, and *Cftr* in MM, 10% FBS, and EF cultures at day 7. Data are presented as mean ± SD (n = 3 wells). One-way analysis of variance on the original expression scale, followed by Dunnett’s multiple-comparisons correction against MM, was used to calculate *P* values. ***P < .001; *****P* < .0001.

**Figure S2. Single adult acinar cells form expandable organoids that retain a window for later acinar redifferentiation.**

**(A)** Representative day-7 images of organoids generated in ECCA, 10% FBS, or EF. *Scale bar:* 500 μm.

**(B)** Colony-forming efficiency under 10% FBS, EF, and ECCA expansion conditions at day 7. Data are presented as mean ± SD (representative experiment with n = 4 wells per condition). One-way analysis of variance was used to calculate *P* values, followed by Dunnett’s multiple-comparisons correction. *****P* < .0001.

**(C)** Quantification of organoid diameter under 10% FBS, EF, and ECCA conditions at day 7. Small points are individual organoids; open symbols are the median of each of three wells and the horizontal line is the mean of the three well medians. Unpaired Welch’s t test on the well medians with Holm correction was used to calculate P values (n = 3 wells) against FBS. *P < .05; ns, not significant.

**(D)** Representative images of expanding organoids at days 1, 3, 5, and 7 in EM. *Scale bar:* 500 μm.

**(E)** Quantification of organoid diameter during expansion. Individual organoid diameters and mouse means are shown (n = 3 mice). Paired t tests on mouse means with Holm correction were used for the four comparisons against day 0. *P < .05; **P < .01; ns, not significant.

**(F)** Immunofluorescence of day-7 organoids stained for Amylase and Krt19, or Pdx1 and Hnf1b, together with the lineage reporter. *Scale bars:* 50 μm.

**(G)** Representative images of limiting-dilution cultures seeded at 4,000, 1,000, 400, or 100 cells per well, shown at day 0, after 7 days of expansion, and after 8 days of redifferentiation. Arrowheads mark tdTomato-negative organoids that remained cystic after transfer to DM. Images are from one seeding series. *Scale bar:* 500 μm.

**(H)** Redifferentiation efficiency of tdTomato-positive and tdTomato-negative organoids after 8 days in DM. Both populations were scored in the same wells and averaged within each mouse (n = 3 mice). Paired t test was used to calculate *P* values. ****P* < .001.

**(I)** Same-field immunofluorescence of a tdTomato-positive and an adjacent tdTomato-negative organoid after 8 days in DM, stained for Amylase together with the lineage reporter and DAPI. Amylase is detected in the tdTomato-positive organoid and is barely detectable in the tdTomato-negative organoid. *Scale bar:* 20 μm.

**(J)** Expression analysis of *Cpa1*, *Hnf1b*, *Mki67*, and *Pdx1* during 7 days of organoid expansion and 10 days of redifferentiation. Data are presented as mean ± SD (n = 3 wells).

**(K)** Representative images of redifferentiated organoids generated from 10% FBS, EF, and ECCA conditions. *Scale bar:* 500 μm.

**(L)** Expression analysis of *Krt19*, *Cftr*, *Amylase*, and *Cpa1* in 10% FBS, EF, and ECCA cultures at day 7 of expansion and day 8 of redifferentiation. Data are presented as mean ± SD (n = 3 wells). Unpaired Welch’s t tests on the original expression scale were used to compare expansion and redifferentiation within each medium, with Holm correction across the three comparisons per gene. **P* < .05; ***P* < .01; ***P < .001; ****P < .0001; ns, not significant.

**Figure S3. Individual ECCA components make distinct contributions to organoid expansion and subsequent redifferentiation.**

**(A)** Schematic overview of culture conditions used for the timed EGF-withdrawal experiments.

**(B)** Schematic overview of culture conditions used for the timed CHIR-99021-withdrawal experiments.

**(C)** Representative images of organoids generated under multiple conditions at day 7. *Scale bar:* 500 μm.

**(D)** Expression analysis of *Amylase*, *Cpa1*, and *Cftr* after organoid expansion under the EGF- and CHIR-99021-withdrawal conditions shown in panel C. Data are presented as mean ± SD (n = 3 wells). One-way analysis of variance was used to calculate *P* values, followed by Dunnett’s multiple-comparisons correction. **P* < .05; ***P* < .01; ****P* < .001; *****P* < .0001; ns, not significant.

**(E)** Colony-forming efficiency under the conditions shown in panel C. Data are presented as mean ± SD (representative experiment with n = 3 wells per condition). One-way analysis of variance was used to calculate *P* values, followed by Dunnett’s multiple-comparisons correction. **P* < .05; ***P* < .01; ****P* < .001; *****P* < .0001; ns, not significant.

**(F)** Quantification of organoid diameter under the conditions shown in panel C. Small points are individual organoids; open symbols are the median of each of three wells and the horizontal line is the mean of the three well medians. Unpaired Welch’s t test on the well medians with Holm correction was used to calculate *P* values (n = 3 wells) against ECCA. ***P* < .01; ns, not significant.

**(G)** Quantification of organoid diameter under the conditions shown in Figure 2C. Small points are individual organoids; open symbols are the median of each of three wells and the horizontal line is the mean of the three well medians. Unpaired Welch’s t test on the well medians with Holm correction was used to calculate P values (n = 3 wells) against EC. *P < .05; **P < .01.

**(H)** Expression analysis of *Amylase* after redifferentiation under the conditions shown in panel C. Data are presented as mean ± SD (n = 3 wells). One-way analysis of variance was used to calculate *P* values, followed by Dunnett’s multiple-comparisons correction. ***P* < .01; ****P* < .001; *****P* < .0001; ns, not significant; N/A, not available.

**(I)** Expression analysis of *Amylase*, *Cpa1*, and *Krt19* after redifferentiation of organoids generated under the conditions shown in Figure 2C. Data are presented as mean ± SD (n = 3 wells). One-way analysis of variance was used to calculate *P* values, followed by Dunnett’s multiple-comparisons correction. ***P* < .01; ****P* < .001; *****P* < .0001; ns, not significant.

**Figure S4. Preserved acinar architecture strengthens post-expansion acinar redifferentiation.**

**(A)** Representative bright-field, tdTomato, and merged images of cluster-derived organoids after 8 days of redifferentiation, showing compact grape-like structures. *Scale bar:* 50 μm.

**(B)** Immunofluorescence of redifferentiated WT cluster-derived organoids stained for Amylase or Krt19 together with the lineage reporter. *Scale bars:* 50 μm.

**(C)** High-magnification immunofluorescence of Amylase together with the lineage reporter and DAPI in freshly isolated acini, in acini after 7 days in MM, and in organoids after 8 days in DM. All three states are shown with identical acquisition and display settings. *Scale bars:* 5 μm.

**(D)** Expression analysis of *Amylase*, *Cpa1*, *Krt19*, and *Cftr* in freshly isolated acini and ECCA-expanded organoids before and after redifferentiation from single acinar cells and acinar clusters. Values are normalized to the matched single-cell EM condition (Single EM = 1). Points represent mouse means; lines and error bars show mean ± SD (n = 3 mice). Colored symbols compare DM with EM within each origin; black brackets compare cluster-derived with single-cell-derived organoids in DM. Paired t tests with Holm correction were used for the three comparisons per gene: redifferentiation versus expansion within each origin, and cluster-derived versus single-cell-derived organoids after redifferentiation. **P* < .05; **P < .01; ****P* < .001; *****P* < .0001; ns, not significant.

**(E)** Representative images of passaged acinar-derived organoids maintaining cystic morphology and tdTomato labeling. *Scale bar:* 500 μm.

**(F)** Quantification of cumulative growth during organoid passaging. Data are presented as mean ± SD (n = 3 wells).

**(G)** Acinar-marker expression before and after redifferentiation across passages, showing weaker redifferentiation at later passages. Data are presented as mean ± SD (n = 3 wells).

**Figure S5. Acinar cells progress along an injury-associated acinar-to-ductal continuum.**

**(A)** UMAP of the integrated scRNA-seq dataset colored by sample.

**(B)** UMAP showing tdTomato expression across the integrated dataset. Color intensity indicates normalized gene expression level.

**(C)** Cell-type composition across samples in the integrated dataset.

**(D)** Dot plot showing canonical marker expression across major clusters. Dot size indicates the percentage of cells expressing each gene, and color indicates normalized gene expression level.

**(E)** UMAP of tdTomato-positive cells in the acinar-lineage-restricted dataset.

**(F)** UMAP feature plots showing acinar, ductal, epithelial, and proliferation markers across the lineage-restricted trajectory. Color intensity indicates normalized gene expression level.

**(G)** Additional scFates analysis of dynamic marker expression along pseudotime.

**(H)** Violin and jitter plots showing pseudotime distribution across sampling time points.

**Figure S6. Ex vivo acinar-lineage conversion maps to conserved ADM and metaplastic regulatory programs.**

**(A)** Reference cell-state landscape from the Ma *et al.* (2022) pancreatitis atlas used for query projection.

**(B)** Projection of ex vivo acinar-lineage cells onto the Ma *et al.* (2022) reference space, colored by in vitro cell state.

**(C)** Time-resolved transferred-label analysis showing the accumulation of ADM, mucinous, metaplastic, and ductalized reference identities from D0 to D7.

**(D)** Ma *et al.* (2022) reference projection with D0–D7 centroid trajectory and per-time-point query overlays, showing progression from acinar toward ADM and mucin/ductal-like reference regions.

**(E)** UMAP feature plots showing expression of *Cpa1*, *Reg3b*, and *Krt19* on the Ma *et al.* (2022) projection. Color intensity indicates normalized gene expression level.

**(F)** Query-to-reference identity mapping showing how ex vivo cell states resolve into Ma *et al.* (2022) labels.

**(G)** Transferred-label composition analysis showing how Acinar, ADM-like, and Ductal-like ex vivo states align with Ma *et al.* (2022) identities.

**(H)** TF-program activity analysis across native cell states in ex vivo, Ma *et al.* (2022), and Schlesinger *et al.* (2020) datasets. The four programs comprise the 17 regulons shown in Figure 4I — 4 acinar, 4 ADM, 4 ductal-like, and 5 ductal — which are listed in Table S6. Program activity was calculated from mean regulon AUCell scores and z-scored within each dataset and program.

**Figure S7. Signaling dynamics along the metaplastic trajectory and identification of redifferentiation conditions.**

**(A)** Pre-ranked GSEA on per-gene Spearman correlation between expression and scFates pseudotime across all detected genes, scored against the MSigDB Hallmark library. Cell-cycle-related gene sets were excluded post hoc. Bars show Normalized Enrichment Score (NES) for terms with FDR ≤ 0.05.

**(B)** Expression heatmap of 13 genes grouped into IGF signaling (*Igf1*, *Igf1r*, *Insr*), GR signaling (*Nr3c1*, *Fkbp5*), and GR downstream targets (*Klf9*, *Ddit4*, *Dusp1*, *Zfp36*, *Nfkbia*, *Sgk1*, *Tsc22d3*, *Per1*). Left: ex vivo acinar-lineage cells binned into 80 pseudotime quantiles (row-z-scored, smoothed); top ribbon indicates cell-type composition. Middle and right: per-cell-type mean expression (row-z-scored) from Ma *et al.* (2022) and Schlesinger *et al.* (2020), respectively.

**(C)** Group-mean expression trajectories (mean ± SEM across genes) of the three categories from (B) across the ex vivo pseudotime (left), Ma *et al.* cell types (middle), and Schlesinger *et al.* cell types (right).

**(D)** Expression analysis of *Amylase*, *Cpa1*, and *Krt19* after adding dexamethasone or PPP back to DM during days 4–8 (experiment 1) or removing either from continuous PPP + dexamethasone (experiment 2). Values are the log2 fold change relative to the control of each experiment. Data are presented as mean ± SD (n = 3 wells). One-way analysis of variance on log2 expression was used to calculate *P* values, followed by Dunnett’s multiple-comparisons correction. *P < .05; ***P* < .01; ****P* < .001; *****P* < .0001; ns, not significant.

**(E)** Gene expression analysis of DM and each single-component dropout after 8 days of redifferentiation. Values are log2 fold changes relative to complete DM within each mouse. Data are presented as mean ± SD (n = 3 mice).

**(F)** Gene expression analysis of MM and each single-component dropout after 7 days of maintenance. Values are log2 fold changes relative to complete MM within each mouse. Data are presented as mean ± SD (n = 4 mice).

**Figure S8. Additional characterization of *KPC* acinar organoid expansion, redifferentiation, and passaging.**

**(A)** Representative morphology of *KPC* organoids at days 1, 4, and 7 of expansion. *Scale bar:* 500 μm.

**(B)** Colony-forming efficiency comparison between ECCA and CCA in *KPC* cultures. Data are presented as mean ± SD (representative experiment with n = 4 wells per condition). Unpaired Welch’s t test was used to calculate *P* values. ns, not significant.

**(C)** Quantification of organoid diameter between ECCA and CCA in *KPC* cultures. Small points are individual organoids; open symbols are the median of each of three wells and the horizontal line is the mean of the three well medians. Unpaired Welch’s t test on the well medians was used to calculate P values (n = 3 wells). ns, not significant.

**(D)** Gene-expression analysis of *Amylase*, *Cpa1*, *Cftr*, *Krt19*, *Mki67*, and *Pdx1* during expansion of *KPC* organoids in ECCA and CCA.

**(E)** Representative images of *KPC* organoids cultured in 10% FBS and EF during expansion and after redifferentiation. *Scale bar:* 500 μm.

**(F)** Expression analysis of *Krt19*, *Cftr*, and *Amylase* in 10% FBS, EF, and ECCA conditions at day 7 of expansion and day 8 of redifferentiation in *KPC* cultures. Data are presented as mean ± SD (n = 3 wells). Unpaired Welch’s t test was used to calculate *P* values. **P* < .05; ***P* < .01; ****P* < .001; *****P* < .0001; ns, not significant.

**(G)** Expression analysis of *Krt19*, *Cftr*, *Pdx1*, and *Mki67* during redifferentiation of *KPC*-ECCA and *KPC*-CCA organoids. Data are presented as mean ± SD (n = 3 wells).

**(H)** Representative images of *KPC* organoids at passages 0, 1, and 5. *Scale bar:* 500 μm.

**(I)** Expression analysis of *Krt19*, *Cftr*, *Cpa1*, and *Amylase* from passage 0 to passage 5 in *KPC* organoids. Data are presented as mean ± SD (n = 3 wells).
